# Supplementary material for: Gramicidin Lateral Distribution in Phospholipid Membranes: Fluorescence Phasor Plots and Statistical Mechanical Model
Source: Int J Mol Sci. 2018 Nov 21;19(11):3690. doi: 10.3390/ijms19113690 (PMC6274966; doi:10.3390/ijms19113690)
Supplement: Supplementary file 1 [file ijms-19-03690-s001.pdf]

SUPPLEMENTARY MATERIALS

Gramicidin Lateral Distribution in DMPC Liposomal Membranes: Fluorescence Phasor Plots and Statistical Mechanical Model

István P. Sugár[1] , Alexander P. Bonanno[2] and Parkson Lee-Gau Chong[2]

[1]Department of Neurology, Icahn School of Medicine at Mount Sinai, New York, New York 10029, United States
[2]Department of Medical Genetics and Molecular Biochemistry, The Lewis Katz School of Medicine at Temple University, Philadelphia, Pennsylvania 19140, United States

Terms of the free energy of the lattice. The terms of the free energy of the lattice in Eq.3 can be calculated as follows. The half of the internal energies of the *s* and *u* state lattice units E_s and E_u are:

$$E_s = (\varepsilon_g^s + \varepsilon_p^s \frac{M}{2})N_s = N_{tot}(\varepsilon_g^s + \varepsilon_p^s \frac{M}{2})X_g^s \qquad \text{(S1)}$$

$$E_u = \varepsilon_g^u (n - N_s) + \varepsilon_p^u \left(\frac{m - N_s M}{2} \right) = N_{tot} \left[\varepsilon_g^u (X_g - X_g^s) + \varepsilon_p^u \left(1 - X_g - X_g^s \frac{M}{2} \right) \right] \qquad \text{(S2)}$$

where ε_g^s is the lateral interaction energy of a gramicidin (located in one of the layer of a rigid cluster) with nearest neighbor condensed lipids plus half of the gramicidin-gramicidin interaction energy between the elements of the gramicidin dimer, ε_g^u is the lateral interaction energy of a gramicidin (located in one of the layer of a fluid unit) with nearest neighbor fluid lipids plus the interaction energy between the gramicidin and fluid lipids (located in the other layer of the fluid unit). ε_p^u is the energy of a phospholipid molecule in the fluid phase. ε_p^s is the average energy of a phospholipid molecule in a rigid cluster.

Half of the interaction energy between the nearest neighbor lattice units, E_i is calculated by assuming periodic boundary conditions [1].

$$E_i = \varepsilon_{uu} N_{uu} + \varepsilon_{us} N_{us} + \varepsilon_{ss} N_{ss} = \frac{z}{2}\left[\varepsilon_{uu} N_u + \varepsilon_{ss} N_s\right] + wN_{us} \approx$$
$$\frac{z}{2}\left[\varepsilon_{uu} N_u + \varepsilon_{ss} N_s\right] + zw\frac{N_u \cdot N_s}{N_u + N_s} = N_{tot}\frac{z}{2}\left[\varepsilon_{uu} X_g^u + \varepsilon_{ss} X_g^s\right] + N_{tot} zw \frac{X_g^u \cdot X_g^s}{X_g^u + X_g^s} \qquad \text{(S3)}$$

where N_{uu}, N_{ss} and N_{us} are the numbers of nearest neighbor lattice units existing in *u-u*, *s-s* and *u-s* state, respectively. ε_{uu}, ε_{ss} and ε_{us} are half of the interaction energy between nearest

neighbor lattice units existing in *u-u*, *s-s* and *u-s* state, respectively, $w = \varepsilon_{us} - (\varepsilon_{uu} + \varepsilon_{ss})/2$ is the cooperativity energy, *z* is the coordination number of the lattice, and $X_g^s = N_s/N_{tot}$ and $X_g^u = N_u / N_{tot}$. In Eq. S3, the number of *u-s* nearest neighbor lattice units, N_{us} is approximated by $zN_u N_s /(N_u + N_s)$ [see Bragg-Williams approximation in [1]]. Here we note that very few models of statistical mechanics have been solved exactly; in most of the cases one has to rely on approximative methods. Among them, the mean-field approximation (such as the Bragg-Williams approximation) is one of the most widely used. The advantage of the mean-field theory is its simplicity and it correctly predicts the qualitative features of a system in most cases [2].

The half of the internal entropies of the *s* and *u* state lattice units S_s and S_u are:

$$S_s = (\sigma_g + \sigma_p^s \frac{M}{2})N_s = N_{tot}(\sigma_g + \sigma_p^s \frac{M}{2})X_g^s \tag{S4}$$

$$S_u = \sigma_g (n - N_s) + \sigma_p^u \left(\frac{m - N_s M}{2} \right) = N_{tot} \left[\sigma_g (X_g - X_g^s) + \sigma_p^u \left(1 - X_g - X_g^s \frac{M}{2} \right) \right] \tag{S5}$$

where σ_g and σ_p^u are the intramolecular entropy of a gramicidin and the intramolecular entropy of a phospholipid molecule in the fluid phase, respectively. Since the gramicidin conformation in the fluid and rigid units are similar [3], the same value of σ_g is taken in the fluid and rigid unit. σ_p^s is the average intramolecular entropy of a phospholipid molecule in a rigid cluster.

The mixing entropy of the molecules of one layer of the bilayer within the fluid phase, i.e. the mixing entropy of the $n - N_s$ gramicidin molecules and the $\left(\frac{m - N_s M}{2} \right)$ phospholipid molecules in the fluid phase, is:

$$S_u^{mix} = kln \binom{n - N_s + \frac{m - N_s M}{2}}{n - N_s} \approx N_{tot} k (X_g - X_g^s) ln \left(\frac{1 - X_g^s (1 + M/2)}{X_g - X_g^s} \right) + N_{tot} k \big(1 - X_g - X_g^s M/2\big) ln \left(\frac{1 - X_g^s (1 + M/2)}{1 - X_g - X_g^s M/2} \right) \tag{S6}$$

where $k = 1.987 cal/(mol \cdot K)$ is the Boltzmann constant, and in the second equality Stirling's approximation [1] was utilized. S_u^{mix} is the sum of the mixing entropies of the *u* state lattice units located in one layer of the bilayer. On the other hand, the mixing entropy of the molecules belonging to an *s* state lattice unit is zero because the gramicidin dimer is assumed to be located at the same place within each rigid cluster and thus the number of microstates within a rigid cluster is 1.

The s and u state lattice units can be arranged along the lattice on $\binom{N_s + N_u}{N_s}$ different ways and thus half of the mixing entropy of the s and u state lattice units is:

$$S_{units}^{mix} = 0.5 \cdot k \cdot ln\binom{N_s + N_u}{N_s} \approx$$

$$0.5 \cdot k \cdot N_s \cdot ln\left(\frac{N_s + N_u}{N_s}\right) + 0.5 \cdot k \cdot N_u \cdot ln\left(\frac{N_s + N_u}{N_u}\right) =$$

$$0.5 \cdot N_{tot} \cdot \left\{k \cdot X_g^s \cdot ln\left(\frac{X_g^s + X_g^u}{X_g^s}\right) + k \cdot X_g^u \cdot ln\left(\frac{X_g^s + X_g^u}{X_g^u}\right)\right\} \quad \text{(S7)}$$

where in the second equality Stirling's approximation [1] was again utilized.

On the energy and entropy parameters of the model. Our model will be applied to gramicidin/DMPC mixtures. Since a lattice unit represents several molecules, the parameters associated with it depend on the actual number of the molecules and their physical and geometrical properties.

Let us consider first the intramolecular energy difference: $\varepsilon_p^s - \varepsilon_p^u$. In an s state lattice unit if the condensing effect of the gramicidin dimer brings all the nearby phospholipid molecules close to the gel state then $\varepsilon_p^s - \varepsilon_p^u \approx -\Delta H_{DMPC} = -10^{-23} kcal$ where ΔH_{DMPC} is the gel-to-fluid transition enthalpy of DMPC bilayers (see Table S1). In reality the average state of the phospholipid molecules in the rigid cluster is between the fluid and gel state. This average state can be characterized by the average of the cross sectional area of the phospholipid molecules in the rigid cluster, $A_{av}(M)$ and thus

$$\varepsilon_p^s - \varepsilon_p^u = -\Delta H_{DMPC} \frac{A_p^f - A_{av}(M)}{A_p^f - A_p^g} \quad \text{(S8)}$$

where

$$A_{av}(M) = \frac{\int_{R_g}^{R(M)} A_p(R) 2R\pi dR}{[R(M)]^2 \pi - R_g^2 \pi} \quad \text{(S9)}$$

and A_p^g is the cross section of a DMPC in gel state.

In Eq.S9, *R(M)* is shown in Figure 7 (red curve) while $A_p(R)$ is given in Fig.7B in [3]. The average cross sectional area of the DMPC molecules in a rigid cluster are plotted against M in Figure S1 (blue curve).

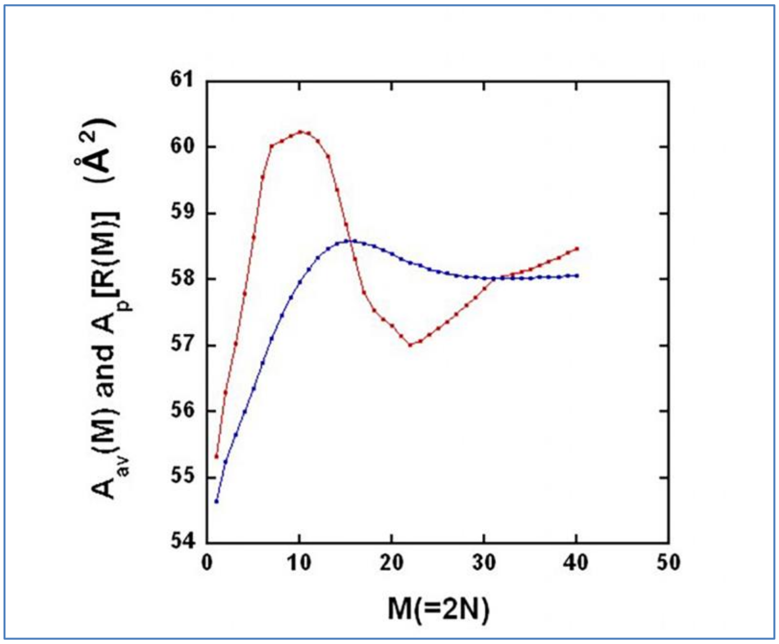

Figure S1. ***Cross sectional area of DMPC molecules in a rigid cluster.*** Blue curve: the average cross sectional area, $A_{av}(M)$ calculated from Eq.S9; red curve: the cross sectional area of a DMPC molecule located at the perimeter of the rigid cluster, $A_p[R(M)]$. $A_p[R(M)]$ is the combination of $A_p(R)$ (given in Fig.7B in [3]) and *R(M)* (shown in Figure 7 (red curve)).

Similar to Eq.S8, one can assume an equation for the intramolecular entropy difference:

$$\sigma_p^s - \sigma_p^u = -\Delta S_{DMPC} \frac{A_p^f - A_{av}(M)}{A_p^f - A_p^g} \qquad \text{(S10)}$$

where ΔS_{DMPC} is the gel-to-fluid transition entropy of DMPC bilayers.

The interaction energy between nearest neighbor lattice units depends on the geometrical and physical properties of the interacting units. Since the size of a unit (either *u* or *s* state) is defined by the size of a rigid cluster, the interaction free energies should be proportional to the circumference or the radius of a rigid cluster, *R(M)*. Thus

$$\varepsilon_{uu} = e_{uu} R(M) \qquad \text{(S11)}$$

$$\varepsilon_{us} = e_{us} R(M) \qquad \text{(S12)}$$

$$\varepsilon_{ss} = \left[e_{ss}^0 + \gamma \left\{ \frac{1}{A_p[R(M)]} - \frac{1}{A_p^f} \right\} \right] R(M) \qquad \text{(S13)}$$

where proportionality factors e_{uu} and e_{us} (related to the *u*-*u* and *u*-*s* interaction energies per unit length, respectively) are constant. However, in the case of *s*-*s* interaction, the value of the proportionality factor changes with the peripheral density of the phospholipid molecules in the rigid clusters. In Eq. S13 the peripheral density is proportional to $1/A_p[R(M)]$ and γ is the proportionality constant. With decreasing *M*, this density increases and the interaction per unit length increases, too. Note that the proportionality factors e_{uu} and e_{us} are considered to be constant because the fluid phase is loosely packed and the short range van der Waals interactions between *u*-*u* and *u*-*s* units are slightly affected by the gramicidin content of the fluid phase. On the other hand, the peripheral density of the rigid clusters depends on the gramicidin content of the rigid phase and has a significant effect on the interaction of the closely packed *s*-units.

Proportion of regularly packed membrane area around $X_{cr}^{11} = 0.154$

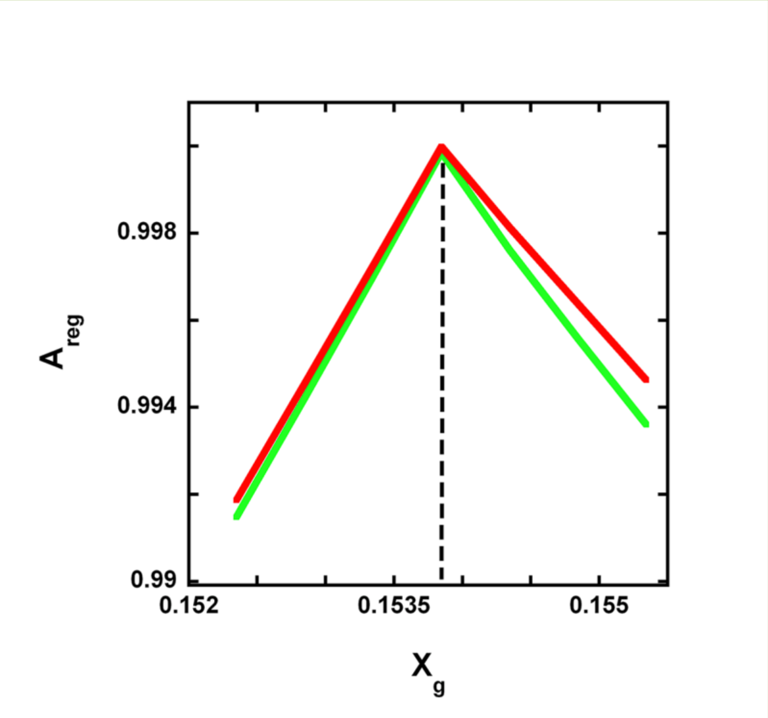

Figure S2. ***Proportion of regularly packed membrane area around*** $X_{cr}^{11} = 0.154$.
Regular area fraction, A_{reg} is plotted against the gramicidin mole fraction, X_g. The model parameters are listed in Table S1 and the energy difference was taken $\varepsilon_g^s - \varepsilon_g^u = 0$ cal/mol (green curve) and $\varepsilon_g^s - \varepsilon_g^u = -1000$ cal/mol (red curve).

List of parameters of the gramicidin/phospholipid model

Table S1. List of parameters of the gramicidin/phospholipid model

**Parameter – symbol**	**Parameter - name**	**Parameter - value**	**Comments and references**
z	Coordination number	4	
A_p^g	Cross sectional area of DMPC in gel phase	40 Å^2	[5]
A_p^f	Cross sectional area of DMPC in fluid phase	62 Å^2	[8]
A_g	Cross sectional area of gramicidin	176.7 Å^2	[6]
$r_g = \left[A_g / \pi\right]^{1/2}$	Radius of gramicidin's cross section	7.5 Å	[6]
A_p^n	cross sectional area of DMPC located next to the gramicidin dimer	53.75 Å^2	[3]
ΔH_{DMPC}	DMPC transition enthalpy	6000cal/mol	[5]
ΔS_{DMPC}	DMPC transition entropy	20.4cal/(mol·K)	[5]
e_{uu}	*u-u* interaction per unit length	-160.5cal/(mol· Å)	[4]
e_{us}	*u-s* interaction per unit length	-207cal/(mol· Å)	[4]
e_{ss}^0	*s-s* interaction per unit length at $M >> 2$	-303.53cal/(mol· Å)	[4]
γ		-10952.48 Å/mol	[4]
T	absolute temperature	310 K	[7]

Note: The values of the cluster-cluster interaction energies per unit length such as e_{uu} , e_{us} , e_{us}^0 and γ were taken from our cholesterol/DMPC model [4] because these phospholipid interaction energies do not change when we switch from cholesterol/DMPC to gramicidin/DMPC mixtures.

<u>Additional phasor data from independently prepared sample sets showing a biphasic change of gD phasor dots at 0.143 gD mole fraction</u>

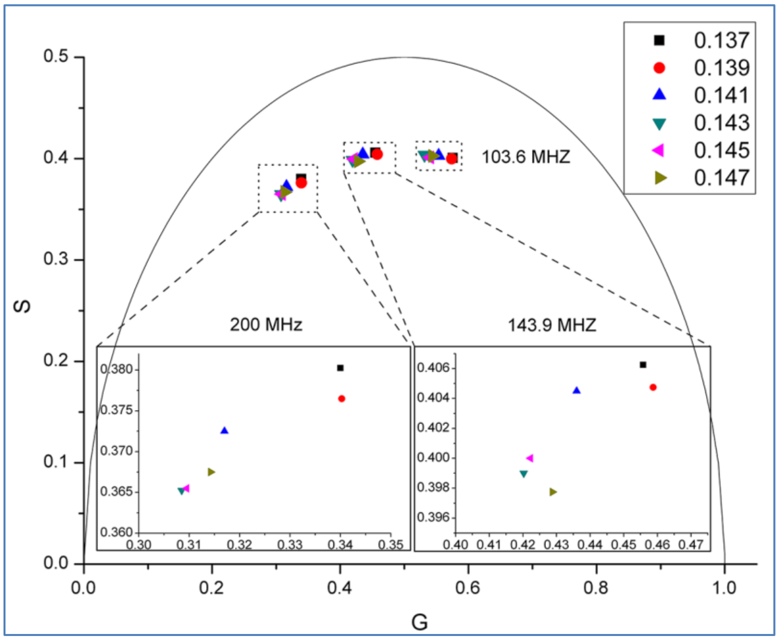

Figure S3. Phasor plot of intrinsic gramicidin fluorescence in various gD/DMPC MLVs with different gD mole fractions ranging from 0.137 to 0.147. This sample set was prepared independently from those sample sets reported in Figures 1-4. Samples were measured at 37ºC using three different modulation frequencies: (from left to right) 200.0, 143.9, and 103.6 MHz. *Inlet*: enlarged phasor data measured at 200.00 and 143.94 MHz. These data indicate that the phasor dot obtained from 0.143 gD mole fraction stands out and is located at left compared to the neighboring non-critical mole fractions, which is consistent with the results reported in Figures 1-4.

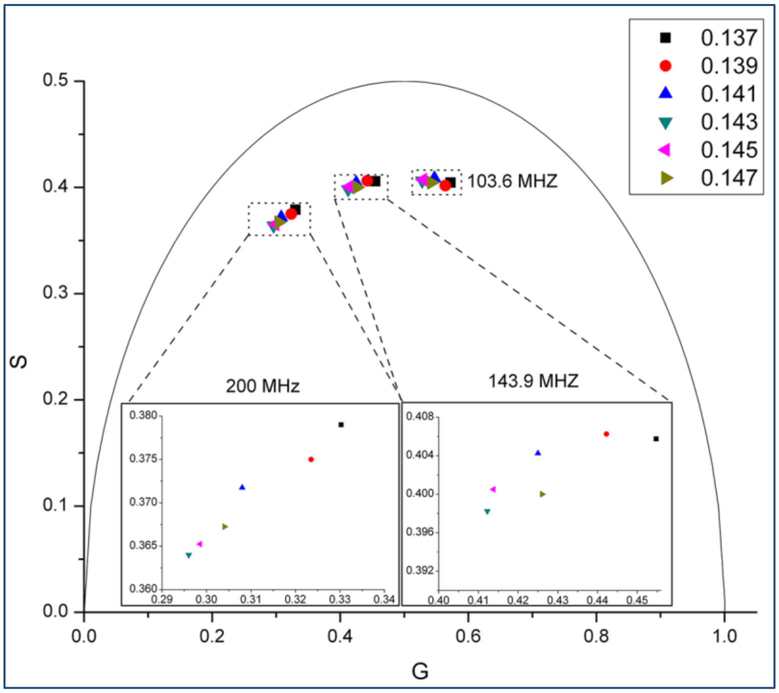

Figure S4. Phasor plot of intrinsic gramicidin fluorescence in various gD/DMPC MLVs with different gD mole fractions ranging from 0.137-0.147. This sample set was prepared independently from those sample sets reported in Figures 1-4 and Figure S3. Samples were measured at 37°C using three different modulation frequencies: (from left to right) 200.0, 143.9, and 103.6 MHz. *Inlet*: enlarged phasor data measured at 200.00 and 143.94 MHz. These data indicate that the phasor dot obtained from 0.143 gD mole fraction stands out and is located at left compared to the neighboring non-critical mole fractions, which is consistent with the results reported in Figures 1-4.

Data showing that there is no biphasic change in lifetime phasor dots of gD intrinsic fluorescence at the critical mole fraction 0.125 gD in DMPC MLVs

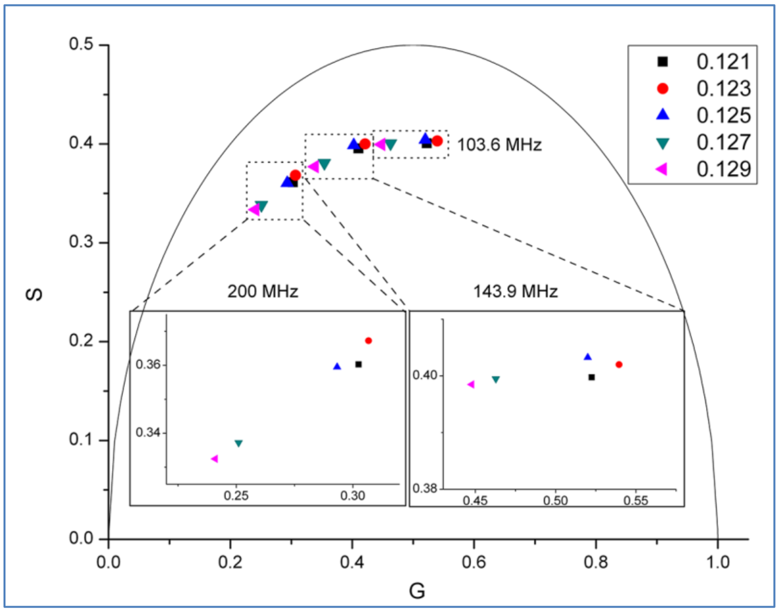

Figure S5. Phasor plot of intrinsic gramicidin fluorescence in various gD/DMPC MLVs with different gD mole fractions ranging from 0.121-0.129. In this sample set, 0.125 is the only theoretically predicted critical mole fraction. Samples were measured at 37°C using three different modulation frequencies: (from left to right) 200.0, 143.9, and 103.6 MHz. *Inlet*: enlarged phasor data measured at 200.00 and 143.94 MHz. These data indicate that there is no biphasic change in lifetime phasor dots at the critical mole fraction 0.125.

Data showing that there is no biphasic change in lifetime phasor dots of gD intrinsic fluorescence at the critical mole fraction 0.133 gD in DMPC MLVs

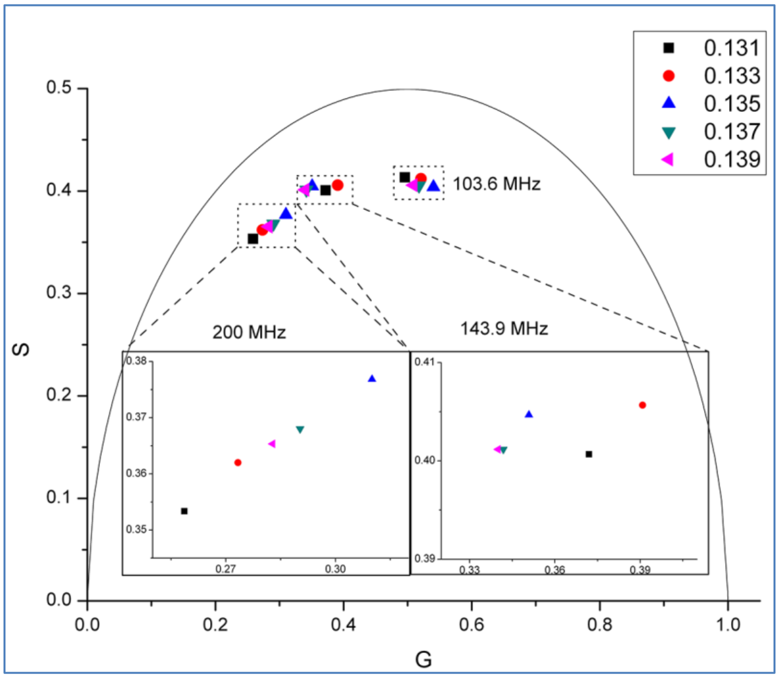

Figure S6. Phasor plot of intrinsic gramicidin fluorescence in various gD/DMPC MLVs with different gD mole fractions ranging from 0.131-0.139. In this sample set, 0.133 is the only theoretically predicted critical mole fraction. Samples were measured at 37°C using three different modulation frequencies: (from left to right) 200.0, 143.9, and 103.6 MHz. *Inlet*: enlarged phasor data measured at 200.00 and 143.94 MHz. These data indicate that there is no biphasic change in lifetime phasor dots at the critical mole fraction 0.133.

REFERENCES

1. Huang, K. *Statistical Mechanics*; Wiley, John & Sons: New York, 1990.
2. Vilfan, I. In Lecture Notes in Statistical Mechanics (2002) The Abdus Salam ICTP, Trieste, Italy and The J. Stefan Institute, Ljubljana, Slovenia, p.73.
3. Kim, T.; Lee, K. I.; Morris, P.; Pastor, R. W.; Andersen, O. S.; Im, W. Influence of Hydrophobic Mismatch on Structures and Dynamics of Gramicidin A and Lipid Bilayers. *Biophys. J* **2012**, *102*, 1551-1560.
4. Sugár, I.P., Chong, L.-G., P. (2012) A statistical mechanical model of cholesterol/phospholipid mixtures: linking condensed complexes, superlattices, and the phase diagram. *J. Am. Chem. Soc. 134,* 1164-1171.
5. Sugár, I.P., Thompson, T.E., Biltonen, R.L. (1999) Monte Carlo simulation of two-component bilayers: DMPC/DSPC mixtures. *Biophys. J.* 76, 2099-2110.
6. Koeppe II, R. E., Berg, J. M., Hodgson, K. O., and Stryer, L. (1979) Gramicidin A crystals contain two cation binding sites per channel. *Nature* 279, 723-725.

7. Wang, M. M.; Sugar, I. P.; Chong, P. L.-G. (1998) Role of the sterol superlattice in the partitioning of the antifungal drug nystatin into lipid membranes. *Biochemistry* 37, 11797-11805.
8. Nagle, J. F.; Tristram-Nagle, S. (2000) Structure of lipid bilayers. *Biochim. Biophys. Acta.* 1469, 159-195.
